# Dysregulation of Stress-Induced Translational Control by *Porphyromonas gingivalis* in Host Cells

**DOI:** 10.3390/microorganisms11030606

**Published:** 2023-02-27

**Authors:** Alex A. Knowles, Susan G. Campbell, Neil A. Cross, Prachi Stafford

**Affiliations:** Biomolecular Sciences Research Centre, Department of Biosciences and Chemistry, Faculty of Health and Wellbeing, Sheffield Hallam University, Sheffield S1 1WB, UK

**Keywords:** *Porphyromonas gingivalis*, Integrated Stress Response, mTOR, gingipains

## Abstract

*Porphyromonas gingivalis* contributes to the chronic oral disease periodontitis, triggering the activation of host inflammatory responses, inducing cellular stresses such as oxidation. During stress, host cells can activate the Integrated Stress Response (ISR), a pathway which determines cellular fate, by either downregulating protein synthesis and initiating a stress–response gene expression program, or by initiating programmed cell death. Recent studies have implicated the ISR within both host antimicrobial defenses and the pathomechanism of certain microbes. In this study, using a combination of immunofluorescence confocal microscopy and immunoblotting, the molecular mechanisms by which *P. gingivalis* infection alters translation attenuation during oxidative stress-induced activation of the ISR in oral epithelial cells were investigated. *P. gingivalis* infection alone did not result in ISR activation. In contrast, infection coupled with stress caused differential stress granule formation and composition. Infection heightened stress-induced translational repression independently of core ISR mediators. Heightened translational repression during stress was observed with both *P. gingivalis*–conditioned media and outer membrane vesicles, implicating a secretory factor in this exacerbated translational repression. The effects of gingipain inhibitors and gingipain-deficient *P. gingivalis* mutants confirmed these pathogen-specific proteases as the effector of exacerbated translational repression. Gingipains are known to degrade the mammalian target of rapamycin (mTOR) and the findings of this study implicate the gingipain-mTOR axis as the effector of host translational dysregulation during stress.

## 1. Introduction

The oral cavity harbors a wide array of biofilm-forming bacteria, which form a symbiotic relationship with their host [1]. However, in some cases, the community becomes dysbiotic with an increased load of pathogenic bacteria, ultimately resulting in oral disease characterized by inflammation of gingival tissues [2,3]. In severe cases, disease progresses into the chronic condition known as periodontitis [3], the sixth most prevalent disease worldwide affecting ~743 million [4]. Periodontal disease has been associated with a range of diseases including cardiovascular disease [5], rheumatoid arthritis [6], diabetes [7], cancer [8], Alzheimer’s disease [9], and Parkinson’s disease [10].

Periodontitis is caused by a variety of pathogenic bacteria, the most prominent pathogens being *Porphyromonas gingivalis*, the keystone pathogen, as well as *Tannerella forsythia* and *Treponema denticola* [2,11]. Invasion of oral epithelial cells by *P. gingivalis* disrupts intracellular homeostasis in several ways [12]. One example is via the major virulence factor gingipains, extracellular cysteine proteases [13], which are known to degrade key host proteins, including the mammalian Target of Rapamycin Complex 1 (mTORC1) [14,15], a protein central to many cellular processes including protein synthesis and autophagy [16]. In addition, *P. gingivalis* inhibits host antimicrobial and phagocytic responses, which can create a favorable replicative niche [12].

Progression of periodontitis leads to an increasingly cytotoxic environment within the periodontal pocket with increasing levels of bacterial metabolites and oxidative stress due to neutrophil activation [17]. Under such stress conditions, host cells can activate several signaling cascades, one of which is a concerted cellular reprogramming system, termed the Integrated Stress Response (ISR), which functions to determine cellular fate [18].

ISR activation initially causes a global downregulation of protein synthesis, which sets out to conserve energy and allow the activation of a stress–response gene expression program thereby allowing cells to overcome the stress [18]. A variety of stresses, including bacterial infection, activate one or more of four stress response kinases; Protein Kinase R (PKR), Protein Kinase R such as ER Kinase (PERK), General Control Nondepressible 2 (GCN2), and Heme Regulated Inhibitor (HRI) (kinases reviewed by Donnelley et al. [19]; bacteria and kinases reviewed in Knowles et al. [20]). Once activated, these stress–response kinases converge upon the phosphorylation of the eukaryotic initiation factor 2 alpha subunit (eIF2α) at serine 51 [19,21]. eIF2α in its GTP–bound form binds the initiator methionyl tRNA, forming the ternary complex, a prerequisite for functional translation initiation [22]. During homeostatic translation, eIF2-GTP is hydrolyzed to eIF2-GDP, following which eIF2-GTP is regenerated by eIF2B, allowing for subsequent rounds of translation initiation [23,24]. Stress-induced eIF2α phosphorylation blocks the ability of eIF2B to regenerate eIF2-GTP resulting in the abrogation of global translation by inhibiting the formation of the active ternary complex [25,26]. Translation may be stalled independently of eIF2α through the eIF4E binding protein 1 (4E-BP1) [27] regulated by mTORC1 [28].

Independent of the upstream stimuli, translational shutoff pathways result in stalled messenger ribonucleoprotein particles (mRNPs), which are aggregated into cytoplasmic foci known as stress granules. These aid the sorting of mRNPs into those which will be degraded, or re-initiation if stress is overcome and translation resumes [29]. Stress granules form within minutes and dissolve at a similar pace [30]. Therefore, owing to the dynamic nature of their existence, ongoing retrograde transport of components along functioning microtubules is required [31].

In the context of infection, viruses have been well documented to dysregulate translational control and ISR function [32]. Recent studies have reported that bacterial species may also target the host translational control machinery and ISR function (Reviewed in Knowles et al. [20]). Several bacteria are known to activate host ISR stress–response kinases upon infection including *Shigella flexneri*, *Salmonella* [33,34], *Pseudomonas aeruginosa* [35], and Shiga toxin *Escherishia coli* (STEC) [36]. *E. coli* is known to decrease the frequency of cells expressing stress granules during exogenous ISR activation [37], whilst during similar conditions, *S. flexneri* infection increases stress granule frequency and alters their composition [38]. The mechanism by which *S. flexneri* manipulates stress granules is not fully elucidated, however, proposed mechanisms include dysregulation of the cellular microtubule network and inhibition of mTORC1, which both function to regulate the movement of certain stress granule components [34,38].

Intracellular *P. gingivalis* have been shown to degrade mTOR in a manner dependent on secreted lysine-specific gingipain [15]. However, when secreted, both the lysine- and arginine-specific gingipains elicit the downregulation of mTOR activity acting through the PI3K-AKT pathway [39]. Furthermore, *P. gingivalis* has been shown to induce activation of the Unfolded Protein Response (UPR) [40], which interlinks with the ISR [41]. These findings, together with the fact that periodontal infection produces stress through inflammation [12,17], suggest that *P. gingivalis* infection may also manipulate the host translational control pathways and stress granule formation. The overall aim of this study was to determine whether *P. gingivalis* dysregulates host translational control during oxidative stress and alters stress granule dynamics.

## 2. Materials and Methods

### 2.1. Reagents

All cell culture reagents unless otherwise stated were from Sigma/Merck Life Science UK LTD (Dorset, UK).

### 2.2. Cell Culture

The oral squamous carcinoma derived cell line (H357) was maintained in Dulbecco’s modified Eagle’s medium (DMEM; Gibco, Fisher Scientific, Loughborough, UK) supplemented with 10% fetal bovine serum (FBS) and 2 mM L-glutamine (Glu) in a humidified environment (5% CO_2_, 37 °C). Cells were passaged when ~75% confluent by trypsinization and cell viability were assessed using trypan blue exclusion method as previously described [15].

### 2.3. Bacterial Strains and Culture

Bacterial strains used in this study include *P. gingivalis* NCTC11834, W50 (ACTC 53978) and the derivative W50 isogenic mutants K1A (*kgp*::*Em*), E8 (*rgpA::Em rgpB::Tet*) [42], and EK18 (*rgpA::Em rpgB::Tet kgp::Chlor*) [15]. All strains used were a kind gift from Professor G. Stafford (School of Clinical Dentistry, University of Sheffield, UK).

*P. gingivalis* were grown and maintained on fastidious anaerobe agar (Lab M, Bury, UK) containing oxylated horse blood (5% *v*/*v*); TCS Biosciences, Buckingham, UK) and supplemented with antibiotics as required under anaerobic conditions (10% CO_2_, 10% H_2_, and 80% N_2_) at 37 °C. Bacteria were subcultured every 3–4 days for maintenance. Throughout this study, bacteria were used to infect cells when no older than 3–4 days old post-subculturing. During infection, it was ensured that *P. gingivalis* were left no longer than necessary out of anaerobic conditions before cell treatment. *P. gingivalis* were grown as liquid cultures in brain heart infusion broth (BHI, Difco laboratories, East Molesey, Surrey, UK) supplemented with yeast extract (0.5% *w*/*v*), hemin (5 μg/mL), vitamin K (0.5 μg/mL), and cysteine (0.1% *w*/*v*). The purity of liquid cultures was confirmed by Gram staining before use.

### 2.4. Bacterial Infection, Oxidative Stress Induction, and Cell Treatments

H357 were seeded at a density of 6x10^4^ cells/cm^2^ on coverslips or at 3.6 × 10^4^ per cm^2^ in tissue culture flasks in DMEM/Glu/FBS, following which cells were incubated (5% CO_2_, 37 °C) and allowed to adhere overnight. After replacement of overnight media with fresh media, cells were challenged with *P. gingivalis* at a multiplicity of infection (MOI) of 100, harvested from solid agar, at the timepoints as detailed below. Oxidative stress was induced using sodium arsenite (SA, 250 μM) which was added for the final 30 min of infection. Cells were also treated with or without ISRIB (200 nM, 30 min), Nocodazole (200 nM, 30 min), Rapamycin (400 nM, t = 1 h), or Lipopolysaccharide (LPS) purified from *P. gingivalis* (NCTC11834; (Sigma/Merck Life Science, UK) at 1, 5, or 10 μg/mL, t = 2 h. Uninfected cells were included as control.

After treatment, for Western blotting, adherent cells were washed with phosphate buffered saline (PBS) before the addition of lysis buffer (PBS supplemented with 10% *v*/*v* PhosStop (Roche, Basel, Switzerland), 10% *v*/*v* complete EDTA-free protease inhibitors, and 0.1% *v*/*v* SDS). Total proteins were extracted using a cell scraper and cell lysates were stored at −80 °C for a minimum of one hour or overnight after which proteins were recovered by centrifugation (17,200× *g*, 14 min, 4 °C) and stored at −80 °C until required. Total protein extracts were quantified using the Qubit™ protein assay (ThermoFisher, Loughborough, UK) according to manufacturer instructions and expression levels of proteins of interest were probed by Western blotting. For immunofluorescence analysis, cells were fixed as detailed below.

### 2.5. Isolation of Crude Preparations of P. gingivalis Outer Membrane Vesicles (OMVs)

Crude preparations of *P. gingivalis* (NCTC11834) OMVs were extracted as previously described [43]. *P. gingivalis* were grown to a late exponential phase overnight in liquid culture as outlined above. The next day, cultures were adjusted to OD_600_ of 1.0 following which they were subjected to centrifugation (8000× *g*, 5 min, 4 °C). The resulting supernatant was filtered and sterilized (0.22 μM) and centrifuged (100,000× *g*, 2 h, 4 °C), after which the supernatant was discarded and the pellet was resuspended in PBS. Protein content was determined as outlined above and the resulting quantified OMVs were used to challenge H357 cells.

### 2.6. Generation of P. gingivalis Conditioned Media and Gingipain Inhibition

To determine the effect of *P. gingivalis* secreted components, H357 cells were infected (MOI of 1:100) as described above after which the conditioned media was recovered and filtered (0.22 μM) to remove bacteria and other particulate matter. Untreated adherent H357 cells were then challenged with the recovered conditioned media for 2 h. For gingipain inhibition studies, oral epithelial cells were challenged with conditioned media supplemented with either leupeptin (0.2 mM) or Na-Tosyl-Lysine Chloromethyl Ketone (TLCK, 0.5 mM) after which total protein was extracted and levels of proteins of interest were probed by Western blotting.

### 2.7. Western Blotting

Total protein extracts were separated by SDS page electrophoresis (4–20% polyacrylamide gradient gels; Bio-Rad, Watford, UK) before transferring to nitrocellulose membranes (Trans-blot Turbo transfer system, Bio-Rad). For blocking, membranes were incubated for 1 h at room temperature in Tris Buffered Saline (TBS; 37 mM NaCl, 20 mM Tris, pH 7.6) containing 0.1% *v*/*v* Tween 20 (TBST) and either bovine serum albumin (5% *w*/*v* BSA) or powdered milk (5% *w*/*v*) before incubation with primary antibodies overnight at 4 °C. Primary antibodies used include: puromycin (1:500; clone 12D10, MABE343, Merck), phosphorylated eIF2ɑ (serine 51) (1:500, 44-728G, Invitrogen, Fisher Scientific), eIF2ɑ (1:500, ab181467, Abcam, Cambridge, UK), G3BP (1:500, ab56574, Abcam), eIF3b (1:500, ab133601, Abcam), phosphorylated p70-S6 Kinase (Threonine 389) (1:200, 108D2, Cell Signaling, Leiden, The Netherlands), phosphorylated 4E-BP1 (Threonine 37/46) (1:200, 236B4, Cell Signaling), ɑ-tubulin (1:500, 2144, Cell Signaling), acetyl-ɑ-tubulin (1:500, 1215, Cell Signaling), GAPDH (1:10,000, G9545, Invitrogen, Fisher Scientific), and GAPDH (1:10,000, PL0125, Invitrogen, Fisher Scientific). Following incubation, membranes were washed with TBST (3 × 5 min) before incubation with the corresponding fluorescent conjugated secondary antibodies for 1 h (1:10,000, Li-Cor, Cambridge, UK). Proteins were visualized with a Li-Cor Odyssey infrared imager (Li-Cor) and quantified using Image Studio Lite software version 3.1.4 (Li-Cor).

### 2.8. Puromycin Incorporation Assay

The relative rates of protein synthesis were determined using the non-radioactive fluorescence–activated surface sensing of translation assay as previously described [44]. Briefly, post-treatment cells were incubated in culture media containing puromycin (91 µM) and emetine (208 µM) for 5 min (5% CO_2_, 37 °C). Cells were then washed twice with PBS containing cycloheximide (355 µM) and total protein was extracted as detailed above following which puromycin uptake was probed by Western blotting.

### 2.9. Immunocytochemistry

Methanol-fixed cells were first washed with PBS supplemented with Tween 20 (0.5% *v*/*v*; PBST) before blocking in PBS supplemented with BSA (1% *w*/*v*) for a minimum of 1 h at room temperature. Cells were incubated with primary antibodies overnight. The following primary antibodies were used: G3BP (1:500, ab56574, Abcam), eIF3b (1:500, ab133601, Abcam), ɑ-tubulin (1:500, ab6161, Abcam), and *P. gingivalis* (1:500, a kind gift from Prof. G. Stafford, University of Sheffield Dental School). After washing with PBST (3 × 5 min), membranes were incubated with corresponding fluorescent Alexa fluor™ conjugated secondary antibodies for one hour at room temperature. Cells were washed with PBST, mounted using ProLong Gold™ antifade mountant containing DAPI (ThermoFisher), and protein localization was visualized using a Zeiss LSM800 microscope (Carl Zeiss, Cambridge, UK). Images were captured using ZenBlue software version 2.6, either a 40x or 63x plan-apochromat oil objective and a laser with a maximum output of 10 mW at 0.2% laser transmission. Stress granule frequencies, area, and co-localization were quantified using the analysis module of Zeiss ZenBlue software (Carl Zeiss).

### 2.10. Statistical Analysis

Significance between groups was analyzed using the StatsDirect software package version 3.3.5 (Statsdirect Ltd., Birkenhead, UK). Data was first subjected to a Shapiro–Wilks test where data was considered parametric if *p* < 0.05. All data was found to be non-parametric. Significance between unpaired groups was determined using a Kruskal–Wallis test, which if significant, was followed by a Conover–Inman post-hoc test. Significance was set at *p* ≤ 0.05; **** *p* ≤ 0.001; ***, *p* ≤ 0.001; **, *p* ≤ 0.01; *, *p* ≤ 0.05.

## 3. Results

### 3.1. P. gingivalis Infection Heightens Translational Repression and Modulates Stress Granule Formation during Exogenous Stress

**Figure 1:**
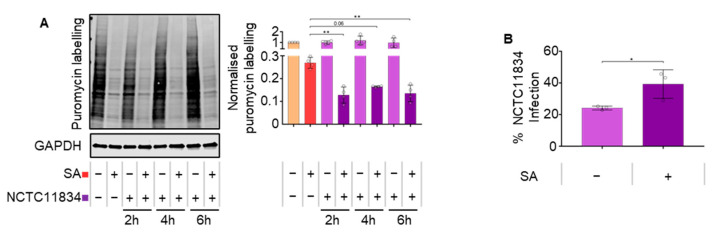
*P. gingivalis* infection heightens translational repression during oxidative stress. (**A**) Relative rate of protein synthesis in infected H357 cells following infection by *P. gingivalis* (NCTC11834, MOI of 100, t = 2–24 h) measured by puromycin uptake (left) and relative quantification by first normalizing to GAPDH and then to untreated sample (mean ± SD, *n* = 4). (**B**) Percentage of H357 cells displaying internalized antibody signal for *P. gingivalis* post-infection (NCTC11834, MOI of 100) after two hours (mean ± SD, *n* = 3). **, *p* ≤ 0.01; *, *p* ≤ 0.05 according to Kruskal–Wallis with Conover–Inman post-hoc.

Bacterial infection can lead to an oxidative stress environment which is known to activate the host-integrated stress–response. To determine if *P. gingivalis* (NCTC11834) can dysregulate the host ISR, the effect on protein synthesis during oxidative stress was monitored in H357 cells. Exposure to Sodium arsenite, a chemical inducer of oxidative stress, had no effect on H357 cell viability. While infection alone did not induce the ISR (Figure S1A–C), a heightened stress-induced translational inhibition of 2.1-fold was observed when cells were treated with both *P. gingivalis* and sodium arsenite (Figure 1A). As this increased inhibition of translation was observed at all infection timepoints, further studies were conducted after 2 h infection.

The effect of oxidative stress on *P. gingivalis* invasion was next determined. In the absence of oxidative stress, *P. gingivalis* infected 24% of cells compared with 39% of total cells in the presence of oxidative stress (Figure 1B).

**Figure 2:**
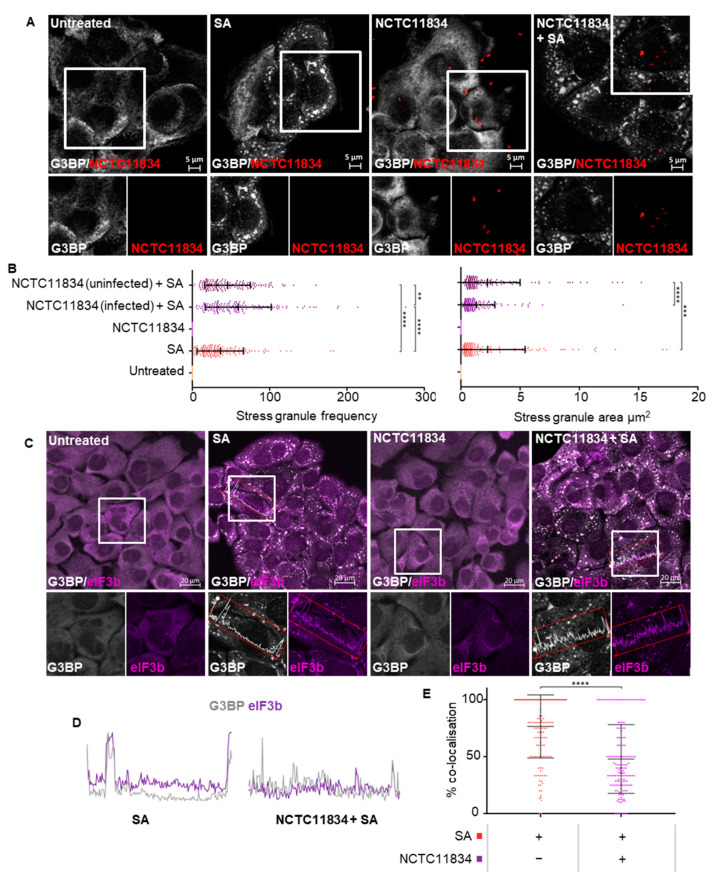
*P. gingivalis* infection modulates stress granule formation during oxidative stress in H357 cells. (**A**) Stress granule formation as visualized by G3BP1 (white) and *P. gingivalis* (red) using confocal microscopy and Z-stacks following *P. gingivalis* (NCTC11834, MOI of 100, t = 2 h) challenge in the presence or absence of sodium arsenite. (**B**) Average area and frequency of stress granules determined in host cells (*n* = 3, 50 cells per biological replicate). (**C**) Co-localization of G3BP1 (white) and eIF3B (purple) in stress granules, as assessed by immunofluorescence. (**D**) Representative line segments of color profiles taken from H357 cells challenged with sodium arsenite with or without *P. gingivalis* (NCTC11834, MOI of 100, t = 2 h) infection, where intensity peaks correspond to stress granules. (**E**) Percentage of colocalization of eIF3b and G3BP1 (*n* = 3, 50 cells per biological replicate). **** *p* ≤ 0.001; ***, *p* ≤ 0.001; **, *p* ≤ 0.01 according to Kruskal–Wallis with Conover–Inman post-hoc.

To establish whether *P. gingivalis* infection could impact the formation of stress granules, the number of stress granules was quantified in cells displaying internalized *P. gingivalis* (NCTC11834) (Figure S1D). Cells treated with oxidative stress induced on average the formation of 36.2 stress granules per cell with an average size area of 2.25 μm^2^. Within the bacteria-treated population, neither uninfected nor infected cells showed evidence of stress granules (Figure 2A). In contrast, when *P. gingivalis* infection was coupled with oxidative stress, the frequency of stress granules increased on average to 59.4 per cell (Figure 2B). Differences between uninfected and infected cells within this population were further characterized and a decrease in stress granules frequency was observed in uninfected cells with the average area (2.2 μm^2^) showing slight variance (Figure 2B).

As stress granule composition is known to be stress-dependent [45], the localization of eIF3b and G3BP in stress granules was analyzed (Figure 2C). During oxidative stress, eIF3b colocalized highly with G3BP positive stress granules (Figure 2D) (mean 75%). However, in the presence of *P. gingivalis* (NCTC11834) and oxidative stress, the mean percentage colocalization of eIF3b to G3BP decreased to 50% (Figure 2D). As *P. gingivalis* is known to degrade several host proteins, the potential for both G3BP or eIF3b degradation was investigated and no degradation was observed (Figure S2), thereby suggesting the ability of *P. gingivalis* to modulate host stress granule frequency and composition.

### 3.2. P. gingivalis Heightens Translational Repression Independently of eIF2α

**Figure 3:**
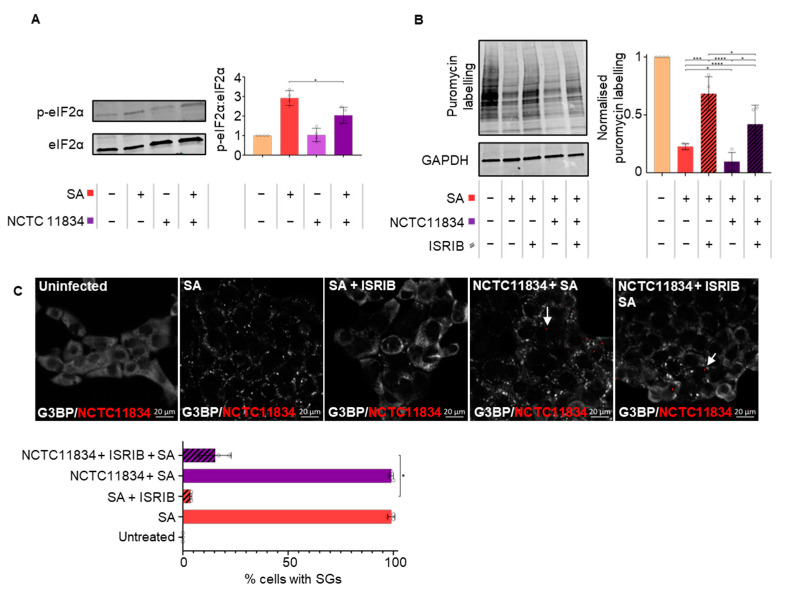
*P. gingivalis* heightens translational repression independently of eIF2α in the presence of stress. (**A**) Level of total and phosphorylated eIF2ɑ (left) and ratio of phosphorylated eIF2ɑ to total eIF2ɑ after *P. gingivalis* (NCTC11834, MOI of 100, t = 2 h) treatment of H357 cells in the presence or absence of sodium arsenite as determined by immunoblotting (right) (mean ± SD, *n* = 4). (**B**) Relative rate of protein synthesis when H357 cells were treated as above and with ISRIB as determined by puromycin uptake (left) and the concentration relative to GAPDH (right) (mean ± SD, *n* = 4). (**C**) Stress granule (SG) formation as visualization of G3BP1 (white) and *P. gingivalis* (red) using confocal microscopy and Z-stacks (*n* = 3, 100 cells per biological replicate). **** *p* ≤ 0.001; ***, *p* ≤ 0.001; *, *p* ≤ 0.05 according to Kruskal–Wallis with Conover–Inman post-hoc.

Translational stalling during stress is classically mediated via the phosphorylation of alpha subunit of eIF2 at serine 51 [19,21]. The relative levels of total and p-eIF2ɑ in *P. gingivalis* (NCTC11834)-infected cells treated with or without oxidative stress were determined by immunoblotting (Figure 3A). A similar basal level of p-eIF2ɑ (Figure 3A) was observed in *P. gingivalis*-infected cells and the untreated control. Strikingly, despite the increased translational repression observed when *P. gingivalis* infection was co-treated with oxidative stress, a decrease in levels of p-eIF2ɑ was observed compared with the oxidative–stress–only treatment (Figure 3A).

The small molecular ISR Inhibitor (ISRIB) is known to reverse the effects of p-eIF2ɑ on translational inhibition and stress granule formation [46]. Here, the ability of ISRIB to attenuate the heightened translational repression and modulation of SG formation during *P. gingivalis* (NCTC11834) infection and oxidative stress was determined. As expected, during ISRIB treatment alone, protein synthesis remained at steady state rates (Figure S3). ISRIB was able to partially rescue stress-induced translation and this rescue was attenuated by *P gingivalis* (Figure 3B). *P. gingivalis* did not affect the proportion of cells containing stress granules (Figure 2B) whereas oxidative stress potently induced stress granule formation, which was inhibited with ISRIB. *P. gingivalis* partially but significantly reversed the ISRIB-induced reduction in stress granules (Figure 3C). Collectively, these data suggest that the heightened translational repression is independent of the ISR and cannot be rescued by ISRIB.

### 3.3. P. gingivalis Heightens Translational Repression via the Action of a Secretory Factor

**Figure 4:**
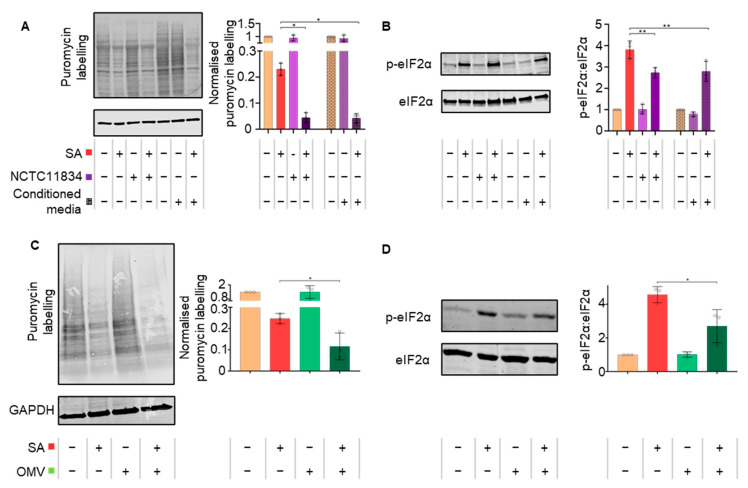
*P. gingivalis* heightens translational repression via the action of a secretory factor. (**A**) Relative rate of protein synthesis as determined by puromycin uptake (left) and quantification relative to GAPDH (right), when H357 cells were treated with filtered conditioned media recovered from cells previously treated with *P. gingivalis* (NCTC11834, MOI of 100, t = 2 h) in the presence or absence of sodium arsenite. (**B**) Levels of phosphorylated eIF2α (left) and concentration of phosphorylated to total eIF2α (right) when probed using immunoblotting (mean ± SD, *n* = 3). (**C**) H357 cells were challenged with purified *P. gingivalis* (NCTC11834) OMV vesicles (100 µg/mL, t = 2 h) with or without sodium arsenite for the final 30 min and the relative rate of protein synthesis measured by puromycin uptake (left) and concentration relative to GAPDH (right) and (**D**) the levels of phosphorylated eIF2α (left) and the concentration of phosphorylated to total eIF2α (right) with OMVs were probed using immunoblotting (mean ± SD, *n* = 3). **, *p* ≤ 0.01; *, *p* ≤ 0.05 according to Kruskal–Wallis with Conover–Inman post-hoc.

As uninfected cells within the infected population displayed increased stress granule frequency during oxidative stress and infection, the effect of *P. gingivalis* (NCTC11834)-conditioned media was investigated to determine whether the observed effects were due to secreted bacterial components. In cells treated with conditioned media and oxidative stress, protein synthesis and eIF2α phosphorylation also decreased in line with infected cells, compared with the oxidative–stress–only treatment (Figure 4A,B). Taken together, these findings demonstrate that factors released by *P. gingivalis* can heighten oxidative stress-induced translational inhibition.

To establish which secreted bacterial constituents elicited the heightened translational inhibition observed during stress, cells were challenged with crude preparations of *P. gingivalis* (NCTC11834) OMVs or purified lipopolysaccharide (NCTC11834 derived LPS). OMVs (1 μg/mL, 10 μg/mL and 100 μg/mL; t = 2 h) did not induce stress (Figure S4A,B). In the presence of oxidative stress, OMVs (100 μg/mL, t = 2 h) heightened translational repression 2.13-fold (Figure 4C) and decreased p-eIF2ɑ 1.69-fold (Figure 4D).

Commercially purified *P. gingivalis* LPS (1, 5 and 10 μg/mL, t = 2 h) did not induce stress (Figure S4C,D). In the presence of oxidative stress, LPS (10 μg/mL, t = 2 h) did not alter translational repression or p-eIF2ɑ (Figure S4E,F). This indicates that the heightened translational repression induced by *P. gingivalis* can be attributed to a secretory component distinct from LPS but present within the isolated OMV fractions.

### 3.4. P. gingivalis Dysregulates mTOR Signaling during Stress

**Figure 5:**
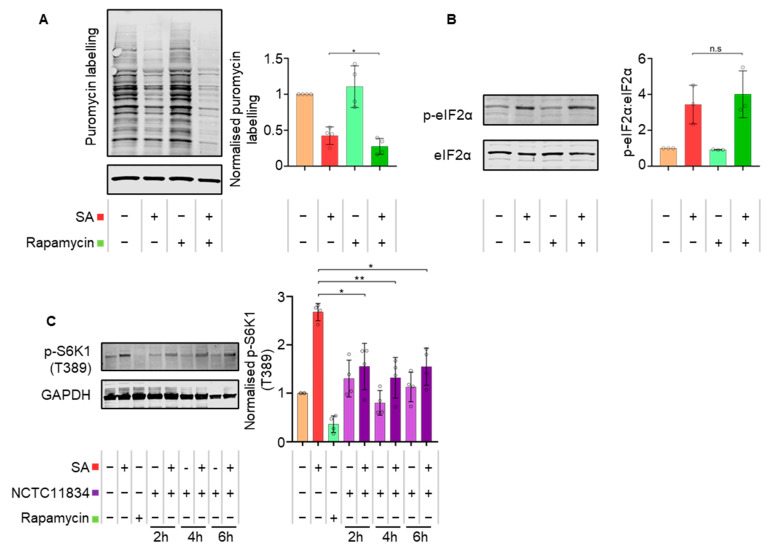
Rapamycin treatments exert the same effect on translation during oxidative stress as *P. gingivalis* and *P. gingivalis* attenuates stress-induced p-p70-S6-Kinase (T389). (**A**) Relative rate of protein synthesis as measured by puromycin uptake (left) and relative concentration compared to GAPDH in H357 cells treated with rapamycin and sodium arsenite as determined by immunoblotting (right). (**B**) Levels of phosphorylated eIF2α (left) and ratio of phosphorylated to total eIF2ɑ as determined by immunoblotting (right) (mean ± SD, *n* = 3). (**C**) Levels of p-p70-S6K1 (T389) (left) and p-p70-S6K1 (T389) concentration relative to GAPDH in cells treated with *P. gingivalis* (NCTC11834, MOI of 100, t = 2–6 h) with and without sodium arsenite as determined by immunoblotting (right) (mean ± SD, *n* = 4). **, *p* ≤ 0.01; *, *p* ≤ 0.05 according to Kruskal–Wallis with Conover–Inman post-hoc.

Upon stress, mTORC1 has also been shown to contribute to translational control [47]. Previously, *P. gingivalis* has been shown to both inhibit and degrade mTORC1 through the activity of its gingipains [15,39]. As heightened translational repression during *P. gingivalis* infection and oxidative stress was independent of eIF2ɑ signaling, the role of mTORC1 was evaluated using the selective mTOR inhibitor, rapamycin (400 nM, t = 1 h). Similar to *P. gingivalis* infection, rapamycin, in the presence of oxidative stress, heightened translational repression (Figure 5A) independently of p-eIF2ɑ (Figure 5B).

To observe the impact of mTOR degradation on translation inhibition during oxidative stress, downstream mTORC1 targets were investigated. Rapamycin decreased the levels of phosphorylated p-p70-S6K1 (T389), whereas oxidative stress induced an increase. In contrast, whilst *P. gingivalis* (NCTC11834) infection alone did not result in altered levels of p-p70-S6K1 (T389), infection in the presence of oxidative stress caused a 1.73-fold decrease at all timepoints investigated (Figure 5C), suggesting that the phosphorylation activity of mTORC1 is downregulated by infection during stress.

### 3.5. Secreted P. gingivalis Proteases, Gingipains, Mediate Heightened Translational Repression during Stress

**Figure 6:**
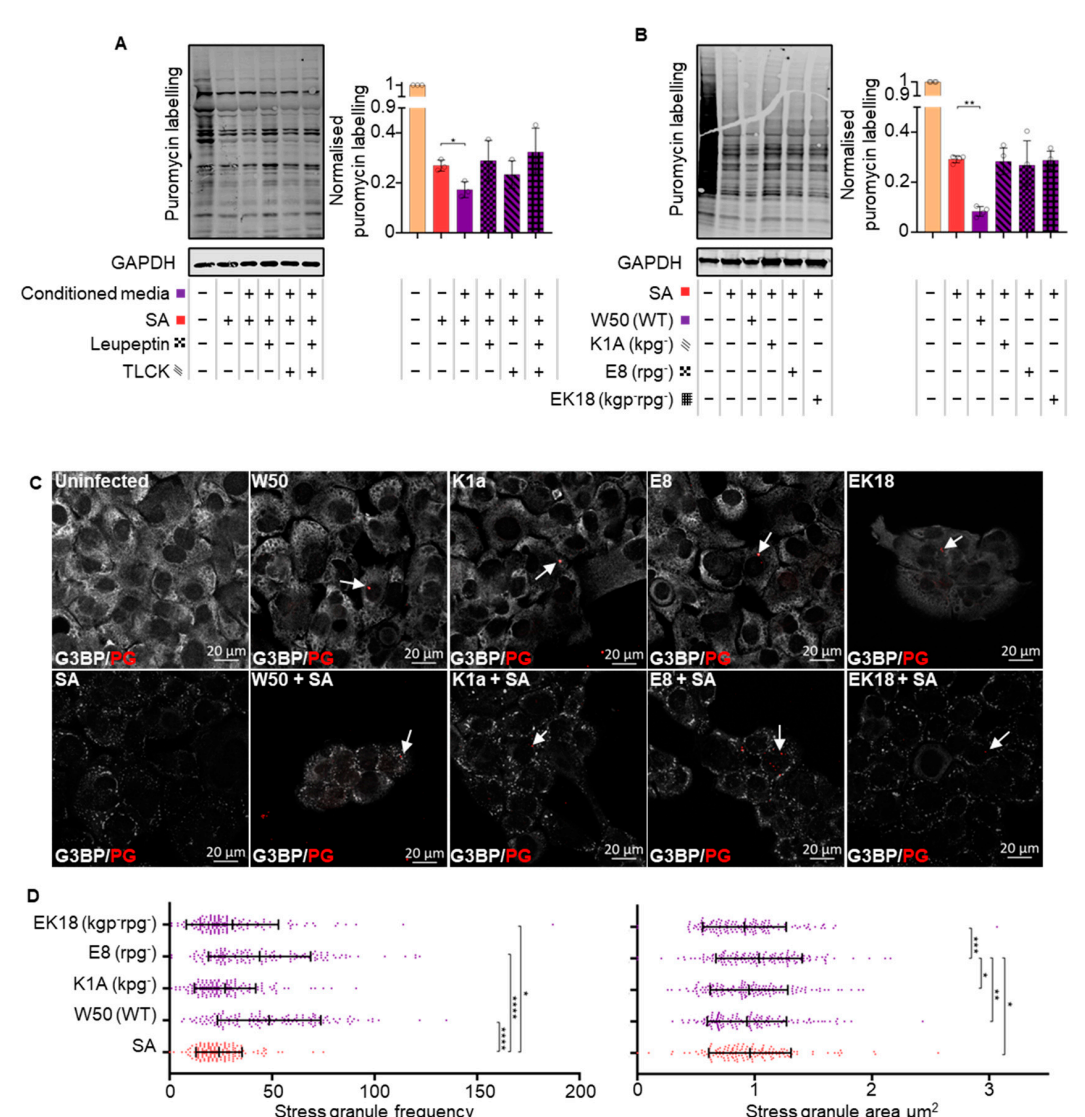
Secreted *P. gingivalis* proteases and gingipains mediate heightened translational repression during infection and stress. (**A**) Relative rate of protein synthesis as determined by puromycin uptake (left) and concentration relative to GAPDH (right) when H357 cells were treated with *P. gingivalis* (NCTC11834)-conditioned media in the presence or absence of leupeptin and TLCK and (mean ± SD, *n* = 3) (**B**) with *P. gingivalis* (W50, K1A (*kgp^−^*), E8 (*rgp^−^*), and EK18 (*rgp^−^kgp^−^*), MOI of 100, t = 2 h); (mean ± SD, *n* = 4). (**C**) Stress granule formation was assessed by visualization of G3BP1 (white) and *P. gingivalis* (red) by confocal microscopy using Z-stacks. (**D**) Average area and frequency of SGs found in cells (*n* = 3, 50 cells per biological replicate). **** *p* ≤ 0.001; ***, *p* ≤ 0.001; **, *p* ≤ 0.01; *, *p* ≤ 0.05 according to Kruskal–Wallis with Conover–Inman post-hoc.

The findings thus far suggest that *P. gingivalis* can heighten translational repression during oxidative stress via a secretory factor. The impact of gingipains on translational control during oxidative stress and infection was therefore probed using gingipain-specific inhibitors TLCK (Lysine-specific, kgp) and Leupeptin (Arginine-specific, rgp). Both Leupeptin and TLCK, either alone or in tandem, inhibited the ability of the *P. gingivalis* (NCTC11834)-conditioned media to heighten translational stalling during oxidative stress (Figure 6A).

To confirm the role of gingipains in translational attenuation, a panel of isogenic gingipain null mutants (K1A, E8 and EK18) in *P. gingivalis* strain W50 were studied. Neither the wild type W50 strain nor the mutants induced a change in protein synthesis during infection in the absence of oxidative stress (Figure S5). Oxidative stress, together with W50, decreased puromycin incorporation (3.5-fold), compared with the oxidative–stress–only treated control. However, the mutants were unable to elicit this phenotype (Figure 6B).

The above data indicate that a role for gingipains in *P. gingivalis* mediated translational repression during oxidative stress. Hence, the effect of gingipains was investigated. During oxidative stress, an increase in stress granules comparable to what was seen with NCTC11834 was observed with wild-type W50 (Figure S6); neither wild-type W50 nor the gingipain mutants induced stress granules or inhibited their formation during oxidative stress (Figure 6C). In an oxidative stress environment, both the wild-type W50 and gingipain mutants E8 and EK18 induced an increase in stress granule frequency which was not observed in K1A infected cells. No change in the average stress granule area was observed with wild-type, K1A, nor EK18, whereas an increase in the area of stress granules was seen with the E8 mutant (Figure 6D). Taken together, these findings suggest that both lysine- and arginine-specific gingipains are accountable for *P. gingivalis*-mediated heightened translation repression during oxidative stress, with the lysine-specific gingipain inducing the increased stress granule frequency.

### 3.6. P. gingivalis Infection Dampens Stress-Induced Tubulin Acetylation

**Figure 7:**
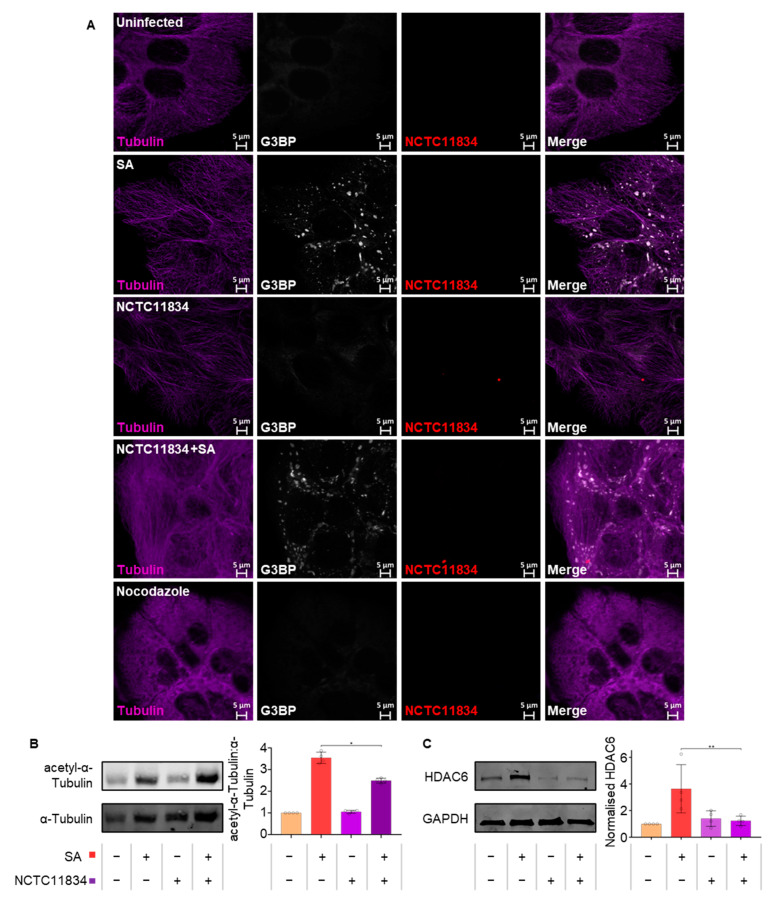
*P. gingivalis* infection dampens stress-induced tubulin acetylation. (**A**) Stress granule, ɑ-tubulin integrity, and *P. gingivalis* were visualized using confocal microscopy following challenge of H357 cells with *P. gingivalis* (NCTC11834, MOI of 100, t = 2 h) in the presence or absence of sodium arsenite. (**B**) Expression levels of ɑ-tubulin (left) and ratio of acetyl-ɑ-tubulin to ɑ-tubulin (right; mean ± SD, *n* = 4). (**C**) Expression of HDAC6 (left) and concentration relative to GAPDH (right; mean ± SD, *n* = 3) as determined by immunoblotting. **, *p* ≤ 0.01; *, *p* ≤ 0.05 according to Kruskal–Wallis with Conover–Inman post-hoc.

As changes to stress granule frequency and area were observed, the integrity of the microtubule network was determined since assembly of mature stress granules requires aggregation of components into smaller foci along polymerizing microtubules [31,48]. Visualization of α-tubulin showed no qualitative changes to the structure of α-tubulin following cell treatment with oxidative stress only or with *P. gingivalis* (NCTC11834) when compared with the total lack of structure observed with the positive control nocodazole (Figure 7A).

As the function of tubulin can be modified post-translationally, the levels of acetyl-α-tubulin were monitored. Both untreated cells and those infected with *P. gingivalis* (NCTC11834) displayed basal level acetylation. Oxidative stress resulted in a 3.6-fold increase in acetylation; a response which was dampened (1.42-fold) when cells were infected with *P. gingivalis* prior to the addition of oxidative stress (Figure 7B).

To investigate the means of tubulin deacetylation during oxidative stress, the expression of the principal tubulin deacetylation enzyme, HDAC6, was determined (Figure 7C). *P. gingivalis* infection (NCTC11834) did not raise HDAC6 above basal levels whilst oxidative stress increased the levels of HDAC6 (3.64-fold). However, this phenotype was not observed when infection was coupled with oxidative stress (Figure 7C), suggesting that the lowered tubulin acetylation observed during *P. gingivalis* and oxidative stress was independent of increased HDAC6 expression.

## 4. Discussion

In recent years, ISR signaling and translational control during stress have garnered increased interest within the remit of host immune responses. These pathways, which can induce a wide variety of outcomes at cellular and systemic levels [49], offer a promising target for pathogens to manipulate. Both bacteria and viruses have been shown to influence the ISR, thereby reprogramming a variety of host responses and enabling the generation of a favorable replicative niche (Reviewed in [20,32]).

This study aimed to investigate the crosstalk between host translational control during stress and *P. gingivalis* infection and the potential wider impact upon periodontal disease progression. It was hypothesized that mTOR degradation following infection may impact translation control during stress, and as *P. gingivalis*-induced mTOR degradation was previously observed identically in both oral squamous carcinoma (H357) and immortalized oral keratinocytes (OK-F6) [15], H357 cells were chosen as the model for these studies.

Previously, *P. gingivalis* infection has been shown to activate the UPR in human umbilical cord endothelial cells [40]. Given that one arm of the UPR feeds into the ISR [41], it was hypothesized that *P. gingivalis* infection may also activate the ISR. However, over a period of 24 h, ISR activation was not observed, as evidenced by a lack of p-eIF2ɑ or translational repression (Figure 1A,B), both core components of the active ISR [18]. Furthermore, infection over the same time did not result in the aggregation of G3BP into stress granules, a downstream marker of translational repression brought on by ISR activity (Figure 1C). Whilst it cannot be formally excluded that these responses might be cell type-specific, with human umbilical cord vein endothelial cells previously used [40] in contrast to the squamous oral epithelial cell carcinoma cells used here, it is possible that the UPR may have been active independent of translational attenuation (discussed further in [50]).

Given that *P. gingivalis* infection alone did not stimulate the ISR, the combined effect of infection and oxidative stress was investigated as reactive oxygen species are produced following neutrophil activation during host inflammatory responses [51]. Sodium arsenite, one of the most well-characterized ISR activating stressors, induces oxidative stress and inflammatory signaling via HRI kinase [52,53] and is known to reliably induce cellular stress [54]. Classically, studies challenge cells with 500 μM sodium arsenite [53,55], however, here, 250 μM (t = 30 min) was used as robust stress–responses without increased cell death as assessed by Hoechst 33342 and propidium iodide staining. The high levels of inflammation characteristic of periodontitis and caused by *P. gingivalis* infection [56] coupled with the expression of oxidative stress resistance genes by *P. gingivalis* [57] made sodium arsenite an attractive and relevant stress.

Previous studies [15,58] and analysis of *P. gingivalis* treated cells here show that 20% of the cells of a population are infected between two and four hours post infection (Figure 1B). In the presence of oxidative stress, bacterial invasion was found to increase 1.6-fold. Although the exact cause of this increase remains to be elucidated, *P. gingivalis* is known to express its own oxidative stress resistance genes [57] and whilst infection initially increases the production of reactive oxygen species [59], *P. gingivalis* later actively protects host cells against reactive oxygen species via the host antioxidant glutathione–response [60]. The layer of hemin present on the surface of *P. gingivalis* acts as a buffer against oxidative radicals and increases *P. gingivalis’* resistance to host oxidative stress [61,62]. *P. gingivalis’* ability to defend against oxidative stress whilst simultaneously upregulating host antioxidant pathways together with sodium arsenite’s ability to decrease mammalian membrane integrity [63] may underpin the increased invasion observed during sodium arsenite induced oxidative stress.

Oxidative stress, as expected, resulted in translational repression [64]. *P. gingivalis*, in the presence of oxidative stress, exacerbated translational repression (Figure 1D) and increased stress granule frequency (Figure 2B). Previous studies looking at *S. flexneri* infection have implicated mTORC1 inhibition due to the membrane damage caused by bacterial internalization in stress granule modulation and translational dysregulation [34,38]. An increase in stress granule frequency has also been reported during chemical mTOR inhibition [65], suggesting that mTORC1 has a role in increased translational attenuation and stress granule frequency. This is supported by the involvement of mTORC1 as a key regulator of translation [66] with its inhibition leading to polysome disassembly and subsequent translational stalling [67]. Although *P. gingivalis* can inhibit and degrade mTOR [15,39], *P. gingivalis* alone did not lead to translational attenuation. A similar result was observed during rapamycin treatment. These differences in translational attenuation could reflect the variable outcomes of mTORC1 inhibition under different conditions [68]. The effects of *P. gingivalis*-mediated inhibition and degradation on translation may therefore only become apparent in the presence of another stress, as seen here where *P. gingivalis* heightened oxidative stress-induced translational attenuation.

Stress granules are formed by sequestration of stalled mRNPs into smaller foci, which in due course, fuse into larger aggregates [48]. The increased frequency of stress granules observed in this study may be due to *P. gingivalis* dysregulating stress granule aggregation and partially excluding eIF3b from the stress granules (Figure 2C,E). This is corroborated by reports that *S. flexneri* can selectively cause delocalization of eIF3b from stress granules during exogenous stress in a manner dependent on mTORC1 inactivation [38]. The movement of eIF3b is regulated by mTORC1, which phosphorylates S6K1 at T389, releasing S6K1 from eIF3b [69]. Oxidative stress-induced p-S6K1 (T389) [70] was decreased by *P. gingivalis* infection (Figure 5C), probably owing to inhibition or degradation of mTORC1 [15,39]. Therefore, decreased p-S6K1 (T389) could account for the lack of eIF3b in stress granules during oxidative stress and *P. gingivalis* infection (Figure 2E) and further supports the role of mTORC1 in the exclusion of eIF3b from stress granules.

Aggregation of stress granules requires constant retrograde transport along functioning microtubules [48,71]. Nocodazole, a chemical which disrupts microtubule assembly, increases the frequency of stress granules [71]. *P. gingivalis* is known to degrade cytoskeletal protein components including β-actin [15,72] and hence, whether the increase in frequency of stress granules observed during infection and oxidative stress was the result of tubulin degradation was determined. Infection did not result in visible changes to the microtubular network compared with nocodazole-treated cells (Figure 7A). However, microtubule network activity can also be controlled via post-translational modifications such as acetylation and phosphorylation [73] with hyper-acetylation of the cellular tubulin network at lysine 40 of α-tubulin reported during stress [74]. α-tubulin hyper-acetylation stimulates increased binding and activity of the microtubule motor proteins dynein and kinesin, which are involved in the movement of stress granules [75,76]. Here, *P. gingivalis* lowered the levels of α-tubulin acetylation during infection and oxidative stress (Figure 7B), which was independent of the increased expression of HDAC6, the major α-tubulin deacetylase [77]. Furthermore, HDAC6 is a critical stress granule component, ablation of which inhibits stress granule assembly [78]. Hence, the decreased tubulin acetylation and lowered HDAC6 expression may be influencing the modulated stress granule formation seen here during *P. gingivalis* infection and oxidative stress.

During ISR activation, translational attenuation, due to a range of stressors, is mediated by the phosphorylation of eIF2α [18]. Dysregulating eIF2α phosphorylation is also a mechanism by which many viruses hijack the host translational function (Reviewed in [79]). In this study, despite infection by *P. gingivalis* heightening translational repression during oxidative stress, no increase in p-eIF2α was observed (Figure 3A). These results were corroborated by the inability of ISRIB, a small molecule which antagonizes the inhibitory effects of p-eIF2 on eIF2B [80], to rescue translational function and to inhibit stress granule assembly during oxidative stress and infection (Figure 3B,C) [46]. Hence, the data points towards a mechanism independent of eIF2α as the mediator of the heightened translational repression seen during *P. gingivalis* infection and oxidative stress.

As the heightened translational repression was eIF2α-independent and downstream mTORC1 targets were altered, rapamycin, a potent mTOR inhibitor, was used to further probe the pathway. During oxidative stress, rapamycin induced the same phenotype as *P. gingivalis* infection and heightened oxidative stress-induced translational stalling independently of p-eIF2α (Figure 5A,B), further supporting the contributory role for mTORC1. These findings are particularly relevant as *P. gingivalis* gingipains, which are known to degrade and inhibit mTOR [15,39], are expressed as cell surface-anchored proteins and in the secretome of *P. gingivalis*, where they exist both freely and packaged within OMVs [13,81]. With *P. gingivalis*-conditioned media and OMVs exhibiting a similar phenotype to internalized bacteria, the role of gingipains was investigated. Inhibition of secreted gingipains in conditioned media by gingipain-specific inhibitors inhibited the heightened translational attenuation observed with the conditioned media (Figure 6A). This inability to induce further translational repression was also observed using gingipain-knockout mutants (Figure 6B). Since secreted gingipains in conditioned media can enter cells in a clathrin-dependent manner [82], these findings implicate both the arginine- and lysine-specific gingipains in an extra- and intracellular manner and is possibly due to mTORC1 inhibition via the PI3K pathway, as reported by Nakayama and colleagues [39]. When the impact of these gingipain-knockout mutants on stress granule formation was investigated, the lysine gingipain-knockout (kgp) failed to increase stress granule frequency (Figure 6D), which could reflect the requirement of intracellular *P. gingivalis* secreted lysine-specific gingipains for mTOR degradation [15]. This may also account for the less marked increase in stress granule frequency in the *P. gingivalis*-negative cells of the exposed population, as OMVs containing gingipains only enter around 8% of cells [83]. In contrast, 40% of cells were infected in this study when exposed to *P. gingivalis* and oxidative stress (Figure 2B). Furthermore, as gingipains are secreted following *P. gingivalis* invasion and internalization of host cells [83,84], it could increase the concentration of intracellular lysine-specific gingipain, compared with conditioned media and OMV treatment. Taken together, these findings suggest that while both gingipains can heighten translational repression, the lysine-specific gingipain is the main effector of stress granule modulation and works most efficiently after invasion.

**Figure 8:**
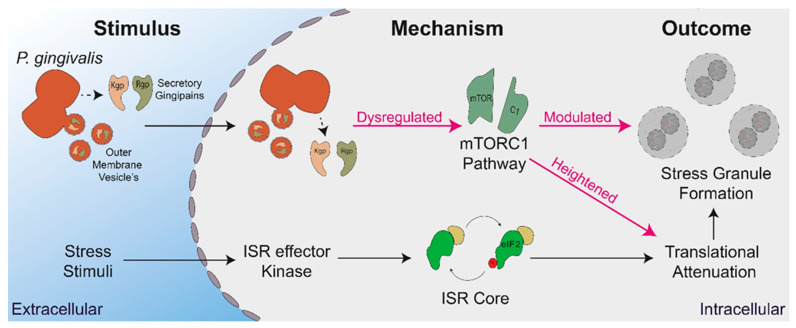
Summary of *P. gingivalis* interactions with host stress-induced translational control. Stress stimuli, such as oxidative stress, activate ISR effector kinases with phosphorylate eIF2ɑ, resulting in translational attenuation and stress granule formation. *P. gingivalis* secretes gingipains and outer membrane vesicles in a both extra- and intracellular manner, which dysregulate the mTORC1 pathway, leading to heightened translational attenuation and modulated stress granule formation.

This study, for the first time, demonstrates that the periodontopathogen *P. gingivalis* dysregulates translational control and stress granule formation during oxidative stress, a condition phenotypic of the chronic inflammatory environment induced during periodontitis and caused by *P. gingivalis* [3] (Illustrated in Figure 8). These findings suggest a novel pathogenic mechanism employed by *P. gingivalis* to modulate host response, given that these pathways feed into cellular survival and the wider immune and inflammatory response [49], can contribute to the immune-subversive nature of *P. gingivalis.* Furthermore, dysregulation of the ISR, translational control, and stress granule dynamics have been implicated in a range of diseases from cancer to neurodegeneration [49,85,86].

While these findings implicate gingipains as defined effectors of translational dysregulation, studies into other *P. gingivalis* virulence factors including capsule, fimbriae, and various effector proteins may also converge onto these pathways. This study utilized a single-cell model and mono-species *P. gingivalis* infection, and to build on and extend these findings, it will be useful to look at the effect of polymicrobial infection on translational control. This is particularly relevant as induction of NOD1 signaling has been reported in periodontic mouse models [87] and NOD1 activation is known to induce ISR activation and NF-κB expression [33]. Furthermore, the impact of such polymicrobial infections of primary cells and 3D tissue models, as well as the effects of long-term infection and chronic stress environments, may offer key insight into the relationship between periodontitis, systemic *P. gingivalis* infection, and other diseases.

## Data Availability

Data sharing is not applicable to this article.

## Supplementary Materials

The following supporting information can be downloaded at: https://www.mdpi.com/article/10.3390/microorganisms11030606/s1, Figure S1. *P. gingivalis* does not induce ISR activation. (A) H357 cells were left untreated, infected with *P. gingivalis* (NCTC11834, MOI of 100, t = 2 h to 6 h) in the presence or absence of sodium arsenite as shown. Relative rate of protein synthesis as measured by puromycin uptake (left) and concentration relative to GAPDH (right) (B) Levels of phosphorylated eIF2α (p-eIF2α) were probed using immunoblotting (left) and the ratio to phosphorylated to total eIF2ɑ was determined (right). GAPDH was included as a loading control (mean ± SD, *n* = 3). (C) Stress granule formation was assessed by visualization of G3BP (white) and *P. gingivalis* (red). (D) H357 cells were infected with *P. gingivalis* (NCTC11834, MOI of 100, t = 24 h). G3BP (white) and *P. gingivalis* (red) were visualized using immunofluorescence confocal microscopy. No significant differences in means were found with a Kruskal–Wallis test. Figure S2. *P. gingivalis* and exogenous stress do not alter G3BP or eIF3B expression. H357 cells were left untreated or infected with *P. gingivalis* (NCTC11834, MOI of 100, t = 2 h to 6 h). Expression levels of (A) G3BP1 and (B) eIF3B were probed using immunoblotting. Concentration relative to the loading control GAPDH was first determined before being normalized to the untreated sample. Data are expressed as mean ± SD, *n* = 3. No significant differences in means were found with a Kruskal–Wallis test. Figure S3. ISRIB treatment does not alter translation in H357 cells. H357 cells were treated with either ISRIB or sodium arsenite, following which the relative rate of protein synthesis normalized first to the loading control GAPDH and then to control sample, was determined by immunoblotting for puromycin incorporation (mean ± SD, *n* = 3). No significant differences in means were found with a Kruskal–Wallis test. Figure S4. *P. gingivalis* outer membrane vesicles and lipopolysaccharide do not induce ISR activation. H357 cells were challenged with purified *P. gingivalis* OMV vesicles (1, 10 and 100 µg/mL, t = 2 h). Sodium arsenite was included as a positive control for ISR activation. (A) The relative rate of protein synthesis as measured by puromycin uptake and (B) the levels of phosphorylated eIF2α were probed using immunoblotting. To the right of each panel is a column graph of the quantified blot signals (mean ± SD, *n* = 2). (C) H357 cells were challenged with purified *P. gingivalis* LPS (1, 5 and 10 µg/mL, t = 2 h). Sodium arsenite was included as a positive control for ISR activation. Following which the relative rate of protein synthesis measured by puromycin uptake and (D) the levels of phosphorylated eIF2α were probed using immunoblotting. To the right of each panel is a column graph of the quantified blot signals (mean ± SD, *n* = 3). GAPDH was included as a loading control. (E) H357 cells were challenged with purified *P. gingivalis* LPS (10 µg/mL) with or without sodium arsenite for the final 30 min and the relative rate of protein synthesis measured by puromycin uptake (left) and concentration relative to GAPDH (right). (F) The levels of phosphorylated eIF2α (left) and the concentration of phosphorylated to total eIF2α (right) with LPS were probed using immunoblotting (mean ± SD, *n* = 3). No significant differences in means were found with a Kruskal–Wallis test. Figure S5. *P. gingivalis* null mutants do not alter protein synthesis. H357 cells were left untreated or infected with *P. gingivalis* (W50, K1A (*kgp^−^*), E8 (*rgp^−^*) and EK18 (*rgp^−^kgp^−^*), MOI of 100, t = 2 h). Relative rate of protein synthesis was measured by immunoblotting for puromycin uptake. GAPDH was included as a loading control (mean ± SD, *n* = 3). No significant differences in means were found with a Kruskal–Wallis test. Figure S6. Comparison of stress granule frequency between NCTC11834 and W50 during stress. H357 cells were left untreated or infected by *P. gingivalis* (strains NCTC11834 and W50, MOI of 100, t = 2 h) and treated with or without sodium arsenite for the final 30 min. Stress granule formation was assessed by visualization of G3BP1 (white) and *P. gingivalis* (red) by confocal microscopy using Z-stacks. (*n* = 3, 50 cells per biological replicate). No significant differences in means were found with a Kruskal–Wallis test.
