# Supplementary material for: Dysregulation of Stress-Induced Translational Control by Porphyromonas gingivalis in Host Cells
Source: Microorganisms. 2023 Feb 27;11(3):606. doi: 10.3390/microorganisms11030606 (PMC10057856; doi:10.3390/microorganisms11030606)
Supplement: Supplementary file 1 [file microorganisms-11-00606-s001.zip › microorganisms-2203633-supplementary.pdf]

Supplementary Materials:

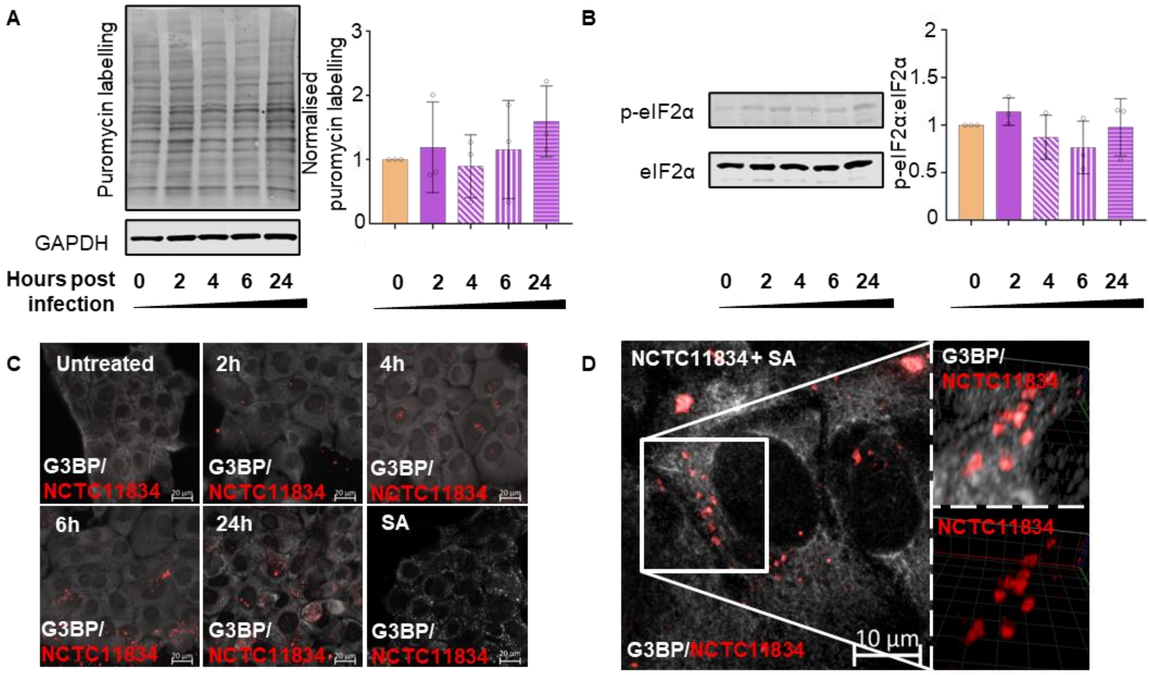

Figure S1. *P. gingivalis* does not induce ISR activation. **(A)** H357 cells were left untreated, infected with *P. gingivalis* (NCTC11834, MOI 1:100, t = 2 h to 6 h) in the presence or absence of sodium arsenite (SA) as shown. Relative rate of protein synthesis as measured by puromycin uptake (left) and concentration relative to GAPDH (right) **(B)** Levels of phosphorylated eIF2*α* (p-eIF2*α*) were probed using immunoblotting (left) and the ratio to phosphorylated to total eIF2α was determined (right). GAPDH was included as a loading control (mean ± SD, *n* = 3). **(C)** Stress granule formation was assessed by visualisation of G3BP (white) and *P. gingivalis* (red). (D) H357 cells were infected with *P. gingivalis* (NCTC11834, MOI 1:100, t = 24 h). G3BP (white) and *P. gingivalis* (red) were visualised using immunofluorescence confocal microscopy. No significant differences in means were found with a Kruskal-Wallis test.

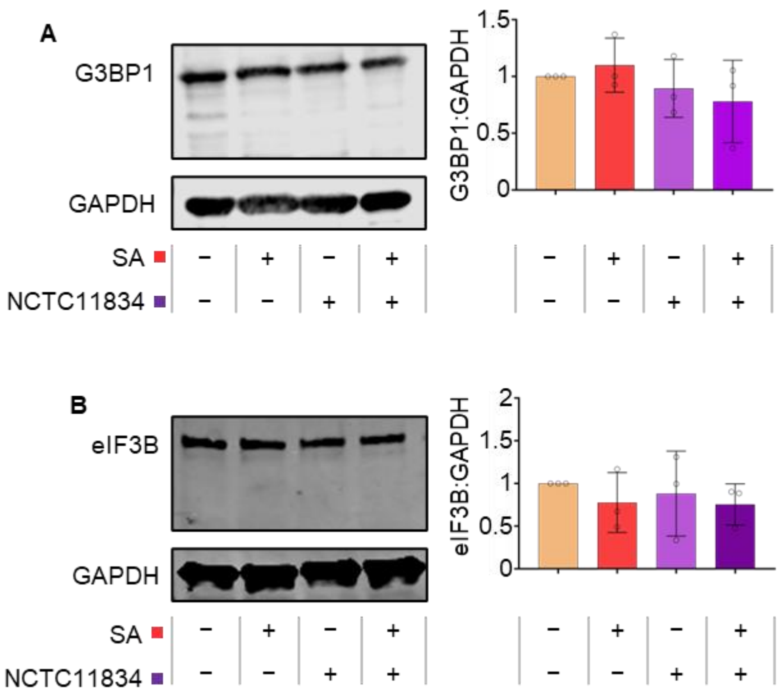

Figure S2. *P. gingivalis* and exogenous stress do not alter G3BP or eIF3B expression. H357 cells were left untreated or infected with *P. gingivalis* (NCTC11834, MOI 1:100, t = 2 h to 6 h), in the presence or absence of sodium arsenite (SA). Expression levels of **(A)** G3BP1 and **(B)** eIF3B were probed using immunoblotting. Concentration relative to the loading control GAPDH was first determined before being normalised to the untreated sample. Data are expressed as mean ± SD, n = 3. No significant differences in means were found with a Kruskal-Wallis test.

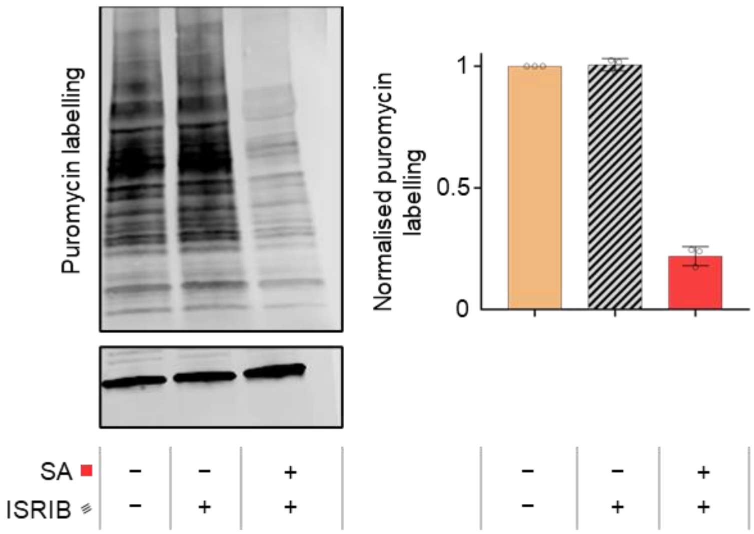

Figure S3. ISRIB treatment does not alter translation in H357 cells. H357 cells were treated with either ISRIB or sodium arsenite (SA), following which the relative rate of protein synthesis, normalised first to the loading control GAPDH and then to control sample, was determined by immunoblotting for puromycin incorporation (mean ± SD, n = 3). No significant differences in means were found with a Kruskal-Wallis test.

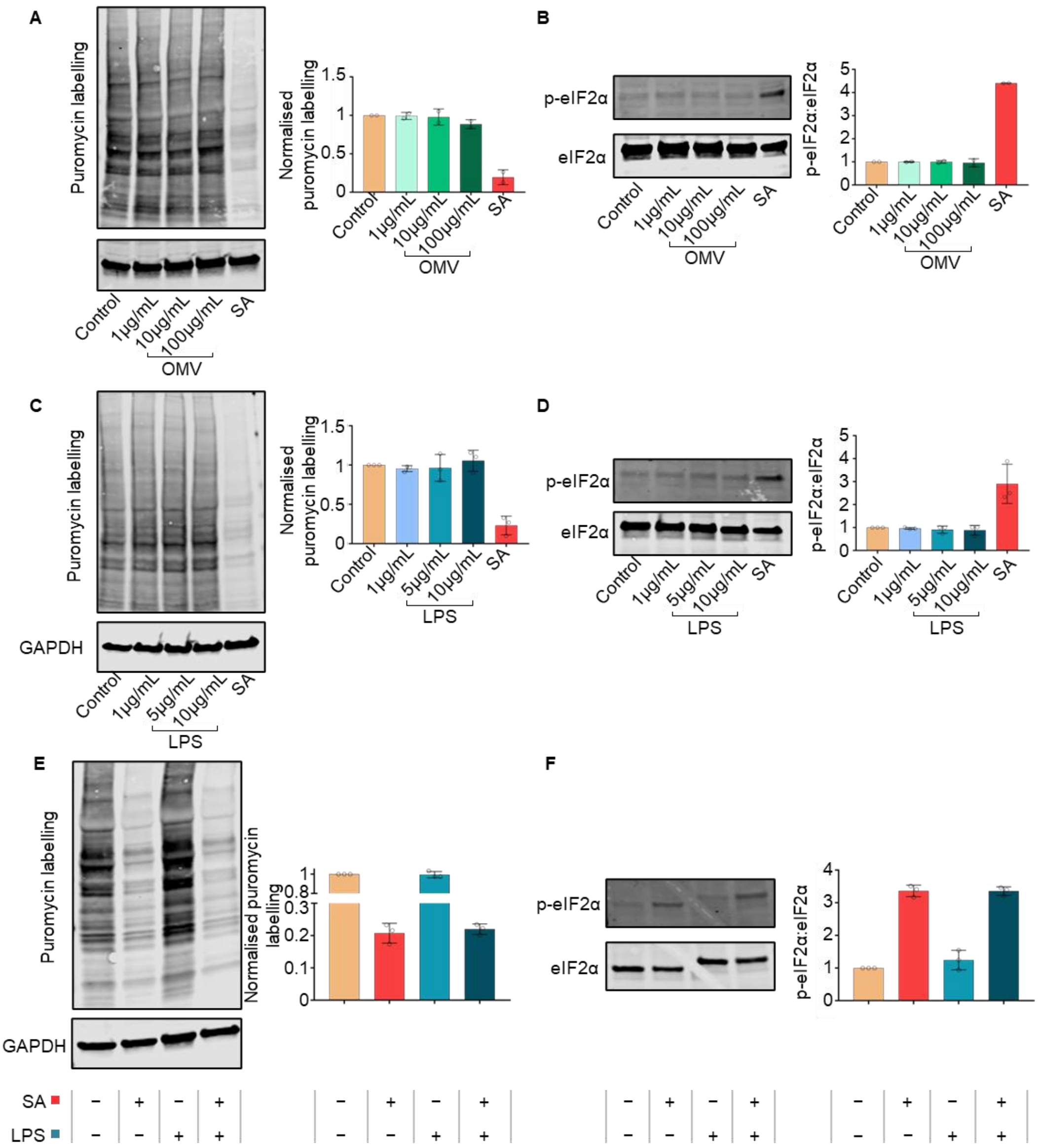

Figure S4. *P. gingivalis* outer membrane vesicles and lipopolysaccharide do not induce ISR activation. H357 cells were challenged with purified *P. gingivalis* OMV vesicles (1, 10 and 100 µg/mL, t = 2 h). Sodium arsenite (SA) was included as a positive control for ISR activation. **(A)** The relative rate of protein synthesis as measured by puromycin uptake and **(B)** the levels of phosphorylated eIF2α were probed using immunoblotting. To the right of each panel is a column graph of the quantified blot signals (mean ± SD, n = 2). **(C)** H357 cells were challenged with purified *P. gingivalis* LPS (1, 5 and 10 µg/mL, t = 2 h). Sodium arsenite was included as a positive control for ISR activation. Following which the relative rate of protein synthesis measured by puromycin uptake and **(D)** the levels of phosphorylated eIF2α were probed using immunoblotting. To the right of each panel is a column graph of the quantified blot signals (mean ± SD, n = 3). GAPDH was included as a loading control. (E) H357 cells were challenged with purified *P. gingivalis* LPS (10 µg/mL) with or without sodium arsenite for the final 30 min and the relative rate of protein synthesis measured by puromycin uptake (left) and concentration relative to GAPDH (right). (F) The levels of phosphorylated eIF2α (left) and the concentration of phosphorylated to total eIF2α (right) with LPS were probed using immunoblotting (mean ± SD, n = 3). No significant differences in means were found with a Kruskal-Wallis test.

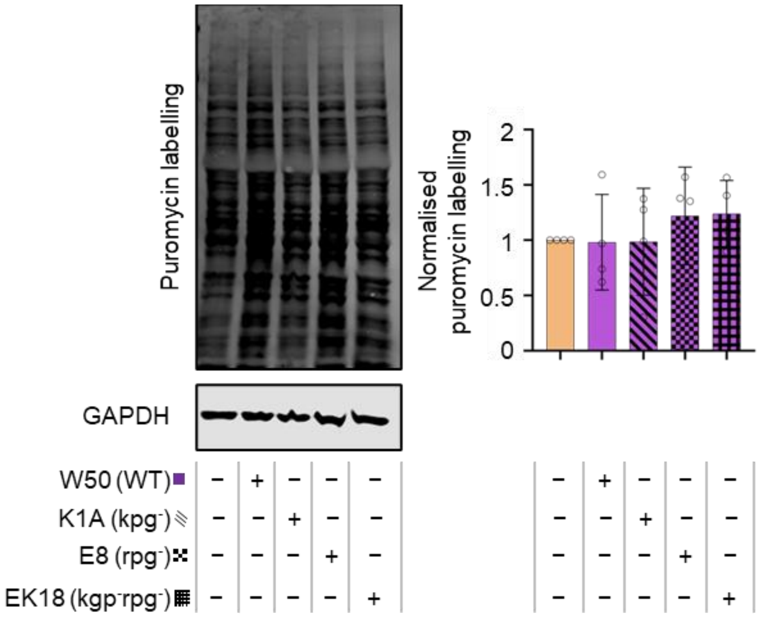

Figure S5. *P. gingivalis null* mutants do not alter protein synthesis. H357 cells were left untreated or infected with *P. gingivalis* (W50, K1A (*kgp*⁻), E8 (*rgp*⁻) and EK18 (*rgp*⁻ *kgp*⁻), MOI 1:100, t = 2 h). Relative rate of protein synthesis was measured by immunoblotting for puromycin uptake. GAPDH was included as a loading control (mean ± SD, *n* = 3). No significant differences in means were found with a Kruskal-Wallis test.

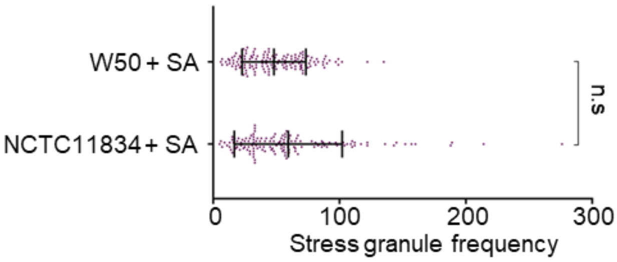

Figure S6. Comparison of stress granule frequency between NCTC11834 and W50 during stress. H357 cells were left untreated or infected by *P. gingivalis* (strains NCTC11834 and W50, MOI 1:100, t=2h) and treated with or without sodium arsenite (SA) for the final 30 min. Stress granule formation was assessed by visualisation of G3BP1 (white) and *P. gingivalis* (red) by confocal microscopy using Z-stacks. (n = 3, 50 cells per biological replicate). No significant differences in means were found with a Kruskal-Wallis test.
